# A Secure Base for Entrepreneurship: Attachment Orientations and Entrepreneurial Tendencies

**DOI:** 10.3390/bs13010061

**Published:** 2023-01-09

**Authors:** Sandra Segal, Mario Mikulincer, Lihi Hershkovitz, Yuval Meir, Tamir Nagar, Yossi Maaravi

**Affiliations:** 1 Adelson School of Entrepreneurship, Reichman University, Herzliya 4610101, Israel; 2 Baruch Ivcher School of Psychology, Reichman University, Herzliya 4610101, Israel

**Keywords:** attachment, entrepreneurial personality, entrepreneurship

## Abstract

Entrepreneurship catalyzes economic growth; it generates jobs, advances the economy and solves global challenges. Hence, it is crucial to understand the factors contributing to entrepreneurship and entrepreneurs’ development. While many studies have investigated intrapersonal factors for entrepreneurial tendencies, the present study focuses on a critical yet often overlooked interpersonal aspect: attachment orientations. Specifically, this article examines the relationship between adult attachment orientations and entrepreneurial tendencies. Three studies across three countries (Israel, the UK, and Singapore) indicated that an anxious attachment orientation in close relationships is negatively associated with enterprising tendencies. In Israel (Study 1) and Singapore (Study 2), avoidant attachment in close relationships was also negatively correlated to such tendencies. Overall, the more people feel secure in close relationships (lower scores on attachment anxiety or avoidance), the higher their enterprising tendencies. Limitations and future research suggestions are discussed.

## 1. Introduction

### 1.1. The Entrepreneurial Personality

There appears to be a consensus that whereas life is becoming increasingly dynamic and uncertain in the 21st century, individuals, organizations, and even countries attribute the entrepreneurial approach to their economic success and prosperity [1]. Kuratko [2] even described it as the “entrepreneurial imperative of the 21st century.” Scholarly interest in entrepreneurship is one manifestation of this attribution (e.g., [3]).

One of the most important avenues of research in entrepreneurship has focused on the contribution of individual-level factors, such as entrepreneurs’ skills, knowledge, tendencies, or traits [4]. Among these individual-level constructs, one can find variables such as proactive personality or need for achievement [5], broader personality constructs (e.g., Big Five traits [6]), self-related beliefs and attitudes (e.g., generalized self-efficacy [7]), and entrepreneurial intentions [8]. Rauch and Frese’s [9] meta-analysis (reviewing a population of 26,700 individuals) summarized the following personality traits characterizing entrepreneurs: need for achievement, generalized self-efficacy, innovativeness, stress tolerance, proactive personality, and need for autonomy. In addition, studies have examined the contribution of internal locus of control [10], opportunity identification [11], and willingness to take risks [12].

Surprisingly, while some broad constructs such as the Big Five traits [6] or generalized self-efficacy [7] have received much scholarly attention, attachment theory [13] has seldom been studied vis-a-vis entrepreneurship. As discussed herein, there is reason to believe that attachment theory may be relevant to examining and understanding individual differences in entrepreneurial tendencies, as it explores the development of emotion regulation, self-regulation, and autonomy in the context of close relationships [13]. Additionally, the entrepreneurship literature has long pointed to the importance of individuals’ familial environment—the breeding ground of attachment—to their personal development as entrepreneurs [14]. Indeed, attachment theory is one of the most influential theories in developmental and personality psychology that can explain individual differences in a wide array of social cognitions and behaviors during childhood, adolescence, and adulthood (e.g., [15]).

The current article explores the relationship between attachment orientations and entrepreneurial tendencies in three studies across three countries: Israel, the United Kingdom, and Singapore. As these three countries differ significantly in their cultural dimensions [16], similarities may suggest a more general relationship between attachment orientations and entrepreneurial personality.

### 1.2. Attachment Theory

Attachment theory, first proposed by Bowlby [13,17], claims that human infants are born with an innate psychobiological system (*the attachment behavioral system*) that motivates them to seek proximity to supportive others (*attachment figures*). This system protects them from physical and psychological threats, and promotes affect regulation, well-being, and healthy autonomy. Bowlby [17] also described critical individual differences in attachment-system functioning that develop as a function of attachment figures’ reactions to a child’s proximity bids in times of need. Interactions with attachment figures who are available and responsive in times of need contribute to the optimal functioning of the attachment system, create positive mental representations of self and others (what Bowlby called *working models* [17]), and promote a sense of attachment security (i.e., a sense that the world is safe and that one can trust others’ goodwill). These positive cognitions allow the person to engage in other non-attachment activities, such as exploring, socializing, or playing, with the confidence that support will be available when needed [18]. However, when a person’s attachment figure is not supportive, a sense of security is not attained, doubts about one’s lovability and others’ intentions ensue, and confident engagement in non-attachment activities is hindered [17]).

Bowlby [19] assumed that the attachment system is active over the entire life span and that working models internalized during interactions with attachment figures solidify into attachment orientations, or chronic patterns of relational expectations, emotions, and behaviors resulting from particular attachment histories [20]. To date, it is well agreed that individual variations in attachment orientations are organized along two roughly orthogonal dimensions of attachment anxiety and avoidance (e.g., [21]). The attachment anxiety dimension indicates the degree to which an individual worries that relationship partners will not be supportive when needed and engages in intensive and coercive attempts to get others’ attention and care. The attachment avoidance dimension indicates the degree to which an individual distrusts others’ intentions and prophylactically maintains emotional distance and independence. Those who score *low* on these two dimensions tend to have a solid sense of attachment security [21].

According to attachment theory, secure attachment, resulting from a history of interactions with available and responsive attachment figures, becomes a resource for resilience and a building block of mental health and social adjustment [22]. Extensive research, including several 20-year longitudinal studies spanning the period from birth to young adulthood (e.g., [23]) has shown that attachment security has beneficial effects on interpersonal behavior functioning, relationship quality, emotion regulation, self-worth, and mental health (see [24] for a review). Recent experimental studies have shown that these findings reflect the action of what Mikulincer and Shaver [25] called the “*broaden-and-build cycle of attachment security*”, a cascade of mental and behavioral events that flow from the activation of mental representations of attachment security to the building of one’s resilience for facing life adversities and the broadening of one’s skills and perspectives. Specifically, contextually activating mental representations of attachment security (using what Mikulincer & Shaver [26] called *security priming* techniques) has positive effects on mood, self-esteem, emotion regulation, mental health, interpersonal functioning, relationship quality, and prosocial feelings and behaviours. Importantly, this occurs even in the case of otherwise insecurely attached people (see [27] for a review).

Due to their adaptive advantages, Bowlby [13] and Ainsworth [18] emphasized the contribution of attachment security to exploration and openness to novelty and uncertainty. According to them, open and relaxed exploration engagement depends upon attachment-system functioning. When an individual feels secure, attachment figures serve as a “secure base” from which s/he can venture out and independently explore novel stimuli. The knowledge that attachment figures will be supportive when needed enables the individual to cope with potential fears and difficulties emerging during the exploration of novel information.

There is extensive evidence supporting the hypothesized attachment-exploration link in adulthood (see [24] for a review). For example, those scoring lower on attachment anxiety or avoidance (higher security) tend to exhibit a greater willingness to engage in social, intellectual, and environmental exploration [28] and innovative leisure activities [29], as well as greater openness to acquiring new information, as manifested in measures of information search, curiosity, and flexibility (e.g., [30]). More secure adults also tend to score lower on cognitive closure, dogmatic and stereotypical thinking, and intolerance of ambiguity (e.g., [30,31]).

### 1.3. Entrepreneurial Tendencies and Attachment

The current study examines whether attachment orientations are associated with entrepreneurial tendencies [32]. In 2012, Schoon and Duckworth [14] showed that parental environment affects the entrepreneurship career path. Specifically, they found that men who had self-employed fathers and women whose parents had socioeconomic resources exhibited more entrepreneurship behavior. Additionally, Zelekha et al. [33] examined the association between attachment orientations and entrepreneurship and found that entrepreneurs scored lower in attachment anxiety than non-entrepreneurs. Moreover, attachment anxiety predicted the intention of becoming an entrepreneur. However, whereas these previous studies show that parental models and attachment orientations are relevant for explaining entrepreneurship intentions and behavior, one crucial question remains: What is the relationship between attachment orientations and entrepreneurial traits? In the current series of studies, we explore a constellation of five specific traits that have been consistently found to distinguish entrepreneurs from non-entrepreneurs and contribute to entrepreneurial success: Need for achievement, need for autonomy, locus of control, risk-taking, and creativity (what Caird [34] called *general enterprising tendency*).

In adulthood, those who have a stronger sense of attachment security have been found to report higher self-esteem [35] and more emotional stability, agreeableness, conscientiousness, openness to differing opinions and new experiences [36], and trust in themselves and others [22]. Moreover, they tend to perform better in creative problem-solving [37] and to feel calmer and more confident when being and doing things alone [38]. Hence, secure attachment appears to lay the foundation for autonomous exploration and inquiry, which is crucial to entrepreneurship [9].

In contrast, more attachment-anxious people tend to suffer more intense and persistent distress, internal doubts, and worries about their lovability and value [15]. Moreover, they have a deep fear of separation and abandonment, find it difficult to be and do things alone, are uncertain about their coping capability, and have fragile self-esteem [22,38], which might prevent the establishment of an independent enterprise [34]. Attachment-avoidant people are more likely to distrust others and to withdraw from social interactions that imply interdependence and cooperation [24], which might hinder the formation and maintenance of a business networking, one of the most critical components of entrepreneurial activity [39]. Moreover, although they prefer to work independently, their compulsive self-reliance [13] leads them to avoid seeking help and thus learning from others, which may impair their entrepreneurial potential [39]. Based on the above research, our central hypothesis is that *lower* scores on attachment anxiety or avoidance scales (more attachment security) will be associated with *higher* entrepreneurial tendencies.

### 1.4. Conceptual Framework: Entrepreneurship as the Ultimate Capacity to Be Alone

While entrepreneurship may be viewed as an occupation, scholars have long referred to it as a personal construct that might be reflected in individuals’ personalities, tendencies, intentions, and behaviors [40]. Indeed, definitions of entrepreneurship and entrepreneurs emphasize their unique exploratory, creative, and proactive approach to life, which leads them to “the discovery, evaluation, and exploitation of opportunities to introduce new goods and services, ways of organizing, markets, processes, and raw materials through organizing efforts that previously had not existed” [41]. Specifically, the entrepreneurial approach involves aspects of risk-taking, responsibility, self-efficacy [42], innovativeness, and proactivity [9], which are all personal capacities that relate to one’s tendency to explore opportunities and seek growth [40].

The ability to explore and investigate one’s environment starts developing in early childhood [43]. The concept of “a holding environment” was suggested by Winnicott [44] to describe an infant’s emotionally and physically supportive environment that promotes its healthy development and, consequently, its transition to autonomy. This experience (which Winnicott [44] termed “going on being”) stems from the presence of a caregiver who is attuned and available to the infant’s needs and refrains from intrusion or neglect [44], which results in the infant recognizing its impulses, feelings, and emotions and thus developing a coherent and continuous self-experience and a sense of emotional security [44]. The child is confident of the caregiver being both a secure base for investigation and exploration of the environment and a safe haven in times of crisis or need [17]. This sense of secure attachment contributes to the foundation of one’s capacities to explore and grow independently.

Children who have experienced a benevolent and reliable caregiver can internalize the caregiver’s comforting and admirable qualities within their evolving selves and consequently feel calm, confident, and strong as they felt while interacting with the caregiver even when s/he is actually absent (known as *incorporation* [45]). In other words, such children feel at peace and relaxed when alone. Eventually, this capacity of “being alone” lays the groundwork for the broader tendencies of curiosity, learning, observing [18], and, therebeyond, openly contemplating, exploring, and creating new ideas [46]. However, when a caregiver is not reliably available or is non-responsive to the child’s needs for a safe haven or secure base, the child might fail to develop the capacity of “being alone” and might interpret times of being alone negatively [38].

In this article, we propose that developing an entrepreneurial personality may rest on an experience of secure attachment. Accordingly, those with a secure working attachment model are not afraid of risk-taking and independent inquiry of opportunities, as they are self-confident and have an established “capacity to be alone”. This, in turn, leads to the ability to reflect on creative ideas, take risks, and flourish in new and uncertain environments, which are part of the entrepreneurial personality [40].

### 1.5. The Current Research

Entrepreneurs operate in an uncertain, pressured environment. Moreover, they are repeatedly required to face failures and refusals from others [47,48]. Additionally, they need a great deal of assistance from various sources, such as investors, colleagues, and mentors [49]. In these cases, an entrepreneur’s sense of safe haven and secure base may act as a “mental immunization” against pressures and failures, promoting and sustaining his or her entrepreneurial tendency.

In contrast, insecure attachment patterns (either anxious or avoidant) tend to interfere with interactions with others and impair a person’s resilience to deal with failures and setbacks [50]. Feeling alone and helpless, attachment-anxious individuals are uncertain about their coping ability, have a fragile self-esteem, and, thus, have a limited ability to initiate activities [22]. Thus, attachment anxiety might inhibit independence and risk-taking, which are necessary for establishing an independent enterprise [34]. Attachment-related avoidance, characterized by distrust of others’ goodwill and inhibition of relational closeness [38], might also compromise entrepreneurial tendencies. Extensive evidence shows that attachment-avoidant individuals tend to feel highly distressed in novel and uncertain situations and lack cognitive openness and willingness to explore and learn, which are essential to entrepreneurship [24]. Overall, whereas insecure patterns might impair entrepreneurial tendencies, a more secure attachment may heighten two core entrepreneurial tendencies: investigation and creativity.

This research aims to examine the effect of attachment patterns on entrepreneurial tendency. This relationship has rarely been studied in the entrepreneurship literature. Therefore, this study’s results may help to understand the kind of relational beliefs (e.g., an inner sense of safe haven and secure base) that sustain entrepreneurship and thus influence public policy to cultivate entrepreneurship.

We tested our hypothesis in three countries. Our first study was conducted in Israel, the second in Singapore, and the third in the United Kingdom. The method and results of each study are presented below. Finally, we discussed this study’s results, implications, and limitations in the Discussion section.

## 2. Study 1

### 2.1. Method

*Participants.* The sample comprised 245 Israelis (112 women; *M*_age_ = 40.62, *SD* = 12.80). Participants were recruited through an Israeli survey agency and responded online. They were paid for their participation.

*Procedure and Measures.* Participants completed scales measuring attachment orientations and enterprising tendencies and a brief demographic questionnaire. The presentation order of the scales was randomized across participants.

Attachment orientations were assessed with the 36-item Experiences in Close Relationships scale (ECR [21]). Participants rated the extent to which each item described their feelings and behaviors in close relationships on a 7-point scale ranging from 1 (*not at all*) to 7 (*very much*). The ECR includes two subscales measuring attachment anxiety and attachment avoidance (18 items per subscale). The reliability and validity of the ECR have been repeatedly demonstrated (beginning with Brennan [21]; see [24] for a review). In the current sample, Cronbach alphas were high for the two ECR subscales: 0.91 for attachment anxiety and 0.89 for attachment avoidance. Mean scores were computed for each participant on each subscale. Higher scores reflect higher levels of attachment anxiety and avoidance.

The enterprising tendency was assessed with the 54-item General Enterprising Tendency questionnaire [42]. This scale consists of five subscales: Calculated risk-taking (e.g., “I like to test boundaries and get into areas where few have worked before”), Need for achievement (e.g., “It is more important to do a job well than to try to please people”), Creative tendency (e.g., “I am wary of new ideas, gadgets, and technologies”), Locus of control (e.g., “ Being successful is a result of working hard; luck has little to do with it “) and Need for autonomy (e.g., “I rarely need or want any assistance and like to put my own stamp on work that I do”). In the current sample, while Cronbach alpha was acceptable for the 54 items (0.73), the alpha coefficients were relatively low when analyzing each of the five subscales separately (αs ranging from 0.34 to 0.59). However, due to the newness of the current study, we tentatively explored the associations of attachment orientations with each of the five subscales beyond examining their association with the total enterprising tendency score. On this basis, we computed a total enterprising tendency score for each participant by summing up answers to the 54 items and five subscale scores by summing up items relevant to each subscale. Higher scores reflect higher enterprising tendencies.

### 2.2. Results and Discussion

**Figure 1:**
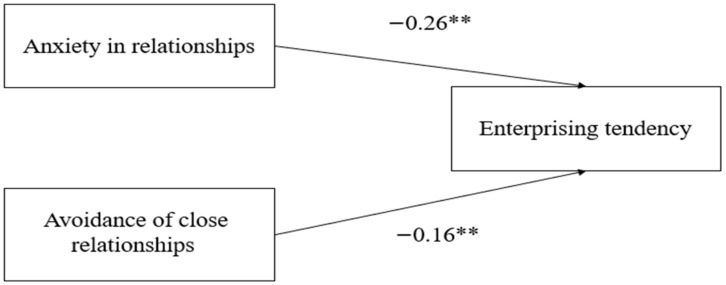
The relationship between attachment patterns and entrepreneurship personality. ** *p* < 0.001. Note: Coefficients are standardized regression weights.

Data were analyzed based on Mikulincer et al.’s [51] multiple regressions for analyzing ECR scores (see Table 1). Specifically, both attachment anxiety and avoidance were entered into a linear regression analysis as predictors, with the total enterprising tendency score as the predicted variable. Both attachment anxiety (β = −0.26, *t* = −4.18, *p* < 0.001) and attachment avoidance (β = −0.16, *t* = −2.69, *p* = 0.008) made a significant unique contribution to the enterprising tendency, *F*(2, 244) = 13.61, R^2^ = 0.10, *p* < 0.001. Consistent with our hypothesis, the lower a participant’s attachment anxiety or avoidance (more secure attachment), the higher her general enterprising tendency (see Figure 1).

As a further exploration, we analyzed each enterprising tendency subscale separately. Specifically, we conducted five multiple regressions with each subscale as the predicted variable and the two attachment dimensions as the predictors. Both attachment anxiety (β = −0.18, *t* = −2.94, *p* = 0.004) and avoidance (β = −0.19, *t* = −2.99, *p* = 0.003) made significant unique contributions to calculated risk-taking, *F*(2, 244) = 9.78, R^2^ = 0.07, *p* < 0.001. Additionally, both attachment anxiety (β = −0.26, *t* = −4.28, *p* < 0.001) and avoidance (β = −0.20, *t* = −3.41, *p* = 0.001) made significant unique contributions to internal locus of control, *F*(2, 244) = 16.64, R^2^ = 0.12, *p* < 0.001. In both cases, attachment insecurities were associated with lower levels of calculated risk-taking and internal locus of control. In addition, attachment anxiety had a significant inverse association with need for achievement, *F*(2, 244) = 5.26, R^2^ = 0.04, *p* = 0.006; β = −0.20, *t* = −3.22, *p* = 0.001), and a marginally significant inverse association with creative tendency, *F*(2, 244) = 2.92, R^2^ = 0.02, *p* = 0.05; β = −0.11, *t* = −1.74, *p* = 0.08. The regression performed on need for autonomy showed no significant contribution to either attachment scores.

This study explored the relationship between attachment and entrepreneurship among Israeli participants. Our results indicate that participants with stronger secure attachment (lower scores in attachment anxiety or avoidance subscales) score higher on the general enterprising tendency. More attachment-anxious participants scored lower in four of the five assessed enterprising tendencies (except for need for autonomy). In contrast, more attachment-avoidant participants scored lower only in calculated risk-taking and internal locus of control. However, these patterns may be culture-specific, as traits such as power distance and uncertainty avoidance have been shown to differ between cultures [16]. Additionally, past research has pointed to cultural differences in entrepreneurship and entrepreneurial tendencies [52,53]. Thus, in order to expand the validity and generalizability of the current study, we attempted to replicate the observed findings in two other cultures: the United Kingdom and Singapore. Based on the relative universality of attachment theory [54], we expected Study 1’s findings to be replicated in these two cultures.

## 3. Study 2

**Figure 2:**
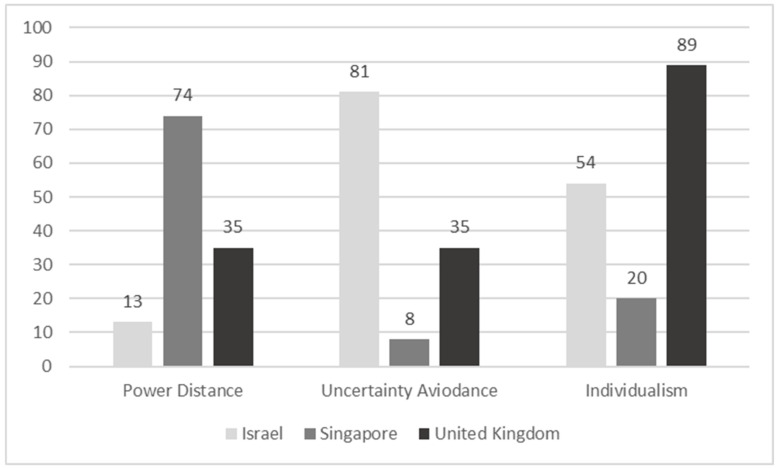
Cultural characteristics relevant to entrepreneurship, according to Hofstede [16], in Israel (Study 1), Singapore (Study 2), and the United Kingdom (Study 3).

Study 2 explored the relationship between attachment patterns and entrepreneurship tendencies in another country, differing in its cultural orientation from Israel. Singapore was chosen as it is an entrepreneurial hub [55], much like Israel, but fundamentally different in its cultural characteristics relevant to entrepreneurship: power distance, uncertainty avoidance, and individualism [16] (see Figure 2).

### 3.1. Method

*Participants.* The sample consisted of 390 Singaporeans (179 women; *M*_age_ = 40.64, *SD* = 10.77). Participants were recruited through an East Asian survey participants recruitment agency. Participants were paid for their participation.

*Procedure and Measures.* The procedure and measures were the same as in Study 1. In Study 2’s sample, Cronbach alphas were high for the two ECR subscales (0.92 for attachment anxiety and 0.79 for attachment avoidance) and acceptable for the total enterprising tendency (0.70), but low for the five enterprising subscales (αs ranging from 0.26 to 0.53).

### 3.2. Results and Discussion

Data were analyzed with the multiple regressions described in Study 1 (see Table 1). Both attachment anxiety (β = −0.16, *t* = −3.21, *p* = 9.001) and attachment avoidance (β = −0.18, *t* = −3.59, *p* < 0.001) made a significant unique contribution to the total enterprising tendency score, *F*(2, 389) = 12.00, R^2^ = 0.06, *p* < 0.001. As in study 1, lower attachment anxiety and avoidance scores (higher security) were associated with higher enterprising tendencies.

Multiple regressions conducted on each enterprising tendency subscale revealed that both attachment anxiety (β = −0.23, t = −4.62, *p* < 0.001) and attachment avoidance (β = −0.10, *t* = −2.01, *p* = 0.04) had significant unique associations with lower levels of internal locus of control, *F*(2, 389) = 13.03, R^2^ = 0.06, *p* < 0.001. In addition, attachment anxiety had a significant unique association with a weaker need for achievement, β = −0.16, *t* = −3.17, *p* = 0.002, *F*(2, 389) = 5.37, R^2^ = 0.03, *p* = 0.005. Moreover, attachment avoidance had a significant unique association with lower levels of calculated risk-taking (β = −0.27, *t* = −5.53, *p* < 0.001, *F*(2, 389) = 16.23, R^2^ = 0.08, *p* < 0.001) and creative tendency (β = −0.15, *t* = −2.97, *p* = 0.003, *F*(2, 389) = 4.56, R^2^ = 0.02, *p* = 0.01). As in Study 1, attachment scores made no significant contribution to the need for autonomy.

Overall, Study 1’s findings from an Israeli sample were replicated in another culture: Singapore. Specifically, in the Singaporean sample, we found that secure attachment (lower scores in attachment anxiety or avoidance) contributed to higher enterprising tendencies. More attachment-anxious participants scored lower in the internal locus of control and need for achievement, whereas more attachment-avoidant participants scored lower in the internal locus of control, calculated risk-taking, and creativity tendencies.

## 4. Study 3

In Studies 1 and 2, the need for autonomy was not associated with attachment orientations. However, neither Israel nor Singapore are individualistic cultures ([16]; see Figure 2). As individualism and the need for autonomy are associated [56], Study 3 aimed to explore the relationship between attachment orientations and entrepreneurial tendencies in general and the need for autonomy in particular in a more individualistic culture: the United Kingdom. Another reason for choosing the United Kingdom was that it is a global entrepreneurial hub, too [55], much like Israel and Singapore.

### 4.1. Method

*Participants*. The sample consisted of 300 British participants (210 women; *M*age = 39.56, *SD* = 11.41) recruited through Prolific, a British study participants recruitment agency. Participants were paid for their participation.

*Procedure and Measures.* The procedure and measures were the same as in Study 1. In Study 3’s sample, Cronbach alphas were high for the two ECR subscales (0.91 for attachment anxiety and 0.89 for attachment avoidance) and acceptable for the total enterprising tendency (0.76), but low for the five enterprising subscales (αs ranging from 0.40 to 0.53).

### 4.2. Results and Discussion

Data were analyzed with the multiple regressions described in Study 1 (see Table 1). Concerning the total enterprising tendency score, the regression model was significant, *F*(2, 297) = 6.55, R^2^ = 0.04, *p* = 0.002). However, only attachment anxiety significantly contributed to the total enterprising score, β = −0.20, *t* = −3.61, *p* < 0.001. Unlike Studies 1 and 2, attachment avoidance made no significant contribution to the total enterprising tendency score, β = 0.02, *t* = 42, *p* = 0.67.

Multiple regressions conducted on each enterprising tendency subscale revealed that attachment anxiety had significant inverse associations with calculated risk-taking (β = −0.18, *t* = −3.17, *p* = 0.002, *F*(2, 297) = 4.93, R^2^ = 0.03, *p* = 0.008) and internal locus of control (β = −0.18, *t* = −3.17, *p* = 0.002, *F*(2, 297) = 8.08, R^2^ = 0.05, *p* < 0.001) and minorly significant association with the need for achievement (β = −0.11, *t* = −1.90, *p* = 0.058, *F*(2, 297) = 2.28, R^2^ = 0.01, *p* = 0.10) and creativity tendencies (β = −0.10, *t* = −1.89, *p* = 0.059, *F*(2, 297) = 2.38, R^2^ = 0.01, *p* = 0.09). Attachment anxiety made no significant contribution to the need for autonomy. Attachment avoidance was significantly associated with an internal locus of control only (β = −0.12, *t* = −2.20, *p* = 0.02.

Finally, after running all three studies, all studies’ data were consolidated. The consolidated data was analyzed with the multiple regressions described in Study 1 with the addition of controlling for possible confounding variables: country, age, gender, and education. Still, after controlling for other variables, both attachment anxiety (β = −0.22, *t* = −6.73, *p* < 0.001) and attachment avoidance (β = −0.12, *t* = −3.90, *p* < 0.001) remain significantly contributing to the total enterprising tendency score: *F*(6928) = 13.20, R^2^ = 0.08, *p* < 0.001. That is, higher security was associated with a higher enterprising tendency. Age (β = −0.005, *t* = −1.77, *p* = 0.87) and education (β = 0.05, *t* = 1.56, *p* = 0.12) did not significantly contribute to the model, whereas country (β = 0.07, *t* = 2.30, *p* = 0.02) and gender (β = −0.10, *t* = −3.15, *p* = 0.002) did. One way ANOVA and Tukey Posy-HOC revealed that Singaporean (M = 29.28, SD = 6.05) had significantly lower enterprising tendency than Israeli (M = 30.89, SD = 6.50; *p* = 0.004) and British (M = 31.14, SD = 6.31; *p* < 0.001). Israeli and British did not significantly differ in their enterprising tendency (*p* = 0.89; F(2932) = 9.02, *p* < 0.001). Additionally, Man (M = 30.86 SD = 6.63) had higher enterprising tendency than women (M = 29.81, SD = 5.98; F(1933) = 6.44, *p* = 0.01).

## 5. General Discussion

As one of the most significant personal and economic growth engines, entrepreneurship leads to human development and prosperity [1]. Therefore, it is crucial to understand the factors contributing to the entrepreneurship phenomenon and the formation of entrepreneurs. Numerous studies have examined entrepreneurs’ traits (e.g., [5,6,7,8]). However, entrepreneurs operate within an ecosystem that includes partners, investors, and mentors. Hence, the *interpersonal* avenue of research is also essential in studying entrepreneurship.

### Conclusions and Implication

The current research aimed to examine one of the most frequently discussed individual-difference factors in the interpersonal domain: adult attachment patterns. Across three studies (in three countries), a secure attachment pattern was positively related to enterprising tendency. Moreover, there was a significant correlation between attachment anxiety and enterprising tendency, so as anxiety in interpersonal relationships decreases, enterprising tendency increases. Finally, the same pattern was found for attachment-related avoidance in Israel (Study 1) and Singapore (Study 2).

The present study contributes to research on both personality development and entrepreneurship and advances our understanding of personality developmental processes and work environment features that can enhance entrepreneurial tendencies and behavior. This research shows the importance of the parental and social environment wherein children grow up and develop entrepreneurial traits such as creativity, risk-taking, and resilience. Furthermore, the current study’s findings highlight the importance of attachment orientations for entrepreneurship and thus enable educators, caregivers, and even decision-makers to design more effective intervention programs and policies for cultivating entrepreneurship.

The current findings strongly emphasize the importance of an individual’s early relationships with primary caregivers in promoting innovative thinking and the ambition to develop and initiate. Moreover, as attachment working models are dynamic throughout the life span, the implications of this research may exceed the parental environment to broader spheres of the environment [57]: family, school, work, culture, economy, and society. Therefore, this research may suggest that countries that wish to promote an entrepreneurial and innovative economy should build circles of influence that will encourage secure attachment among their citizens. For example, implementing intervention programs in which educators will learn to give students a sense of safe haven and secure base or teaching mentors and supervisors in the workplace about the importance of secure attachment at work may not only enhance the tremendous benefits of secure attachment for individuals but may have a positive and significant impact on entrepreneurship and innovation in society itself. We propose a conceptual framework according to which entrepreneurial tendencies rely partly on the experience of a holding and attuned environment that results in an authentic and coherent self [44]. Moreover, this assimilation of a secure base [17,45] may enhance one’s ability to explore and grow independently, known as “the capacity of being alone” [38,44]. This capacity, in turn, is hypothesized to shape and influence abilities to explore, create, and reflect that are crucial to entrepreneurship.

The current research is not without limitations. Below, we describe the main limitations and the future studies we propose to overcome them and further enhance this intriguing avenue of research. First, as seen in Table 1, the associations of attachment scores with entrepreneurship tendency, locus of control, and need for autonomy were identical in the three assessed countries. However, Table 1 also indicates that the pattern of associations of attachment scores with calculated risk-taking, need for achievement, and creative tendency somewhat differ between the three countries. For example, anxious and avoidant attachment in Israel had a negative association with calculated risk-taking; in Singapore, this association was observed only for avoidant attachment, and in the UK, only for anxious attachment. Unfortunately, our data did not allow us to present valid interpretations for these cross-cultural differences as we did not incorporate core cultural variables [16] into our model and analysis. These variables, combined with attachment orientations and entrepreneurial tendencies, may help us to develop a more comprehensive model.

Second, we assessed attachment orientations through self-report scales and did not collect data from qualitative attachment-related interviews that can provide a richer and deeper picture of participants’ attachment history and working models. However, these interviews are highly demanding and expensive and cannot be carried out in large-sample studies that collect information across different countries. Therefore, we decided to use the most reliable and valid self-report scale for assessing attachment orientations. Accordingly, our measure of enterprising tendency is limited in two main ways: It is a self-report questionnaire [58] that measures people’s entrepreneurial tendencies without assessing their entrepreneurial behavior or success. Moreover, we used a single questionnaire [42] that covers only five personality traits, while research has indicated several other personal characteristics that may be relevant (e.g., [5]).

Third, past research has long established the centrality of self-efficacy (both general and context-specific) in the making of entrepreneurs (e.g., [7]). In addition, attachment research has also established the relationship between attachment orientations and several aspects of self-efficacy (e.g., [59]). Future research should investigate the moderating or mediating role that self-efficacy plays in the interplay between attachment and entrepreneurial tendencies. Fourth, research has pointed to several other environmental variables—such as justice, religion, or networks [60]—that may be crucial to understanding the relationship between personality traits and entrepreneurial tendencies and behavior. Fifth, in all three studies, the need for autonomy, which is suggested as a critical element of enterprising tendency [34], was not related to attachment patterns. It is possible that secure attachment, which reflects a balance of togetherness and aloneness [38], differs somewhat from what is measured in Caird’s [34] subscale of *need for autonomy* (a preference to act alone). However, further research using a different methodology is needed to understand this relationship better.

In conclusion, the current study examined the relationship between attachment patterns in adulthood and what may be a manifestation of the “ultimate capacity of being alone”: entrepreneurship. The similar pattern of results across participants from three culturally different countries is an encouraging sign of the proposed relationship between attachment styles and enterprising tendencies.

## Figures and Tables

**Table 1:** Standardized Regression Coefficients of Entrepreneurial Tendencies as a Function of Attachment Anxiety and Avoidance in Each of the Three Assessed Countries.

	Israel		Singapore		United Kingdom	
Attachment orientation ⇨ Entrepreneurial tendencies ⇩	Anxiety	Avoidance	Anxiety	Avoidance	Anxiety	Avoidance
Enterprising Tendency	β = −0.26 ***	β = −0.16 **	β = −0.16 ***	β = −0.18 ***	β = −0.14 *	ns
Calculated Risk−Taking	β = −0.18 **	β = −0.19 **	ns	β = −0.27 ***	β = −0.16 *	ns
Locus of Control	β = −0.26 ***	β = −0.2 ***	β = −0.23 ***	β = −0.1 *	β = −0.13 *	β = −0.10 ^ns^
Need for achievement	β = −0.2 ***	ns	β = −0.16 **	ns	ns	ns
Creative Tendency	β = −0.11 ^ns^	ns	ns	β = −0.15 **	ns	ns
Need for Autonomy	ns	ns	ns	ns	ns	ns

Notes: * *p* < 0.05, ** *p* < 0.01, *** *p* <0.001. ns refers to non-significant.

## Data Availability

The data presented in this study will be openly available at https://osf.io/3hz7j/?view_only=250d30de13214b16a6d2e1b8b87dd268, accessed on 27 December 2022.
